# University students’ perspectives towards digital mental health: a qualitative analysis of interviews from the cross-country ‘CAMPUS study’

**DOI:** 10.1192/j.eurpsy.2024.339

**Published:** 2024-08-27

**Authors:** I. Riboldi, A. Calabrese, S. Piacenti, C. A. Capogrosso, S. Lucini Paioni, F. Bartoli, G. Carrà, J. Armes, C. Taylor, C. Crocamo

**Affiliations:** ^1^Department of Medicine and Surgery, University of Milano-Bicocca , Monza, Italy; ^2^Division of Psychiatry, University College London, London; ^3^Faculty of Health and Medical Sciences, School of Health and Sciences, University of Surrey, Guildford, United Kingdom

## Abstract

**Introduction:**

Poor mental health of university students is a growing concern for public health. Indeed, academic settings may exacerbate students’ vulnerability to mental health issues. Nonetheless, university students are often unable to seek mental health support due to barriers, at both individual and organisational level. Digital technologies are proved to be effective in collecting health-related information and in managing psychological distress, representing useful instruments to tackle mental health needs, especially considering their accessibility and cost-effectiveness.

**Objectives:**

Although digital tools are recognised to be useful for mental health support, university students’ opinions and experiences related to such interventions are still to be explored. In this qualitative research, we aimed to address this gap in the scientific literature.

**Methods:**

Data were drawn from “the CAMPUS study”, which longitudinally assesses students’ mental health at the University of Milano-Bicocca (Italy) and the University of Surrey (United Kingdom). We performed detailed interviews and analysed the main themes of the transcripts. We also performed a cross-cultural comparison between Italy and the United Kingdom.

**Results:**

Across 33 interviews, five themes were identified, and an explanatory model was developed. From the students’ perspective, social media, podcasts, and apps could be sources of significant mental health content. On the one hand, students recognised wide availability and anonymity as advantages that make digital technologies suitable for primary to tertiary prevention, to reduce mental health stigma, and as an extension of face-to-face interventions. On the other hand, perceived disadvantages were lower efficacy compared to in-person approaches, lack of personalisation, and difficulties in engagement. Students’ opinions and perspectives could be widely influenced by cultural and individual background.

**Conclusions:**

Digital tools may be an effective option to address mental health needs of university students. Since face-to-face contact remains essential, digital interventions should be integrated with in-person ones, in order to offer a multi-modal approach to mental well-being.

**Disclosure of Interest:**

None Declared

